# Supplementary material for: The Mediating Role of Psychological Well-Being in the Relationship between Self-Care Knowledge and Disease Self-Management in Patients with Hypertensive Nephropathy
Source: Int J Environ Res Public Health. 2022 Jul 12;19(14):8488. doi: 10.3390/ijerph19148488 (PMC9317263; doi:10.3390/ijerph19148488)
Supplement: Supplementary file 1 [file ijerph-19-08488-s001.zip › ijerph-1782014-supplementary.pdf]

Reporting checklist for cross sectional study.

Based on the STROBE cross sectional guidelines.

Instructions to authors

Complete this checklist by entering the page numbers from your manuscript where readers will find each of the items listed below.

Your article may not currently address all the items on the checklist. Please modify your text to include the missing information. If you are certain that an item does not apply, please write "n/a" and provide a short explanation.

Upload your completed checklist as an extra file when you submit to a journal.

In your methods section, say that you used the STROBE cross sectionalreporting guidelines, and cite them as:

von Elm E, Altman DG, Egger M, Pocock SJ, Gotzsche PC, Vandenbroucke JP. The Strengthening the Reporting of Observational Studies in Epidemiology (STROBE) Statement: guidelines for reporting observational studies.

		Reporting Item	Page Number
**Title and abstract**			
Title	#1a	Indicate the study's design with a commonly used term in the title or the abstract	P1
Abstract	#1b	Provide in the abstract an informative and balanced summary of what was done and what was found	P1-P2
**Introduction**			
Background / rationale	#2	Explain the scientific background and rationale for the investigation being reported	P4-P8
Objectives	#3	State specific objectives, including any prespecified hypotheses	P7-P8

Methods

Study design	#4	Present key elements of study design early in the paper	P8
Setting	#5	Describe the setting, locations, and relevant dates, including periods of recruitment, exposure, follow-up, and data collection	P8
Eligibility criteria	#6a	Give the eligibility criteria, and the sources and methods of selection of participants.	P8
	#7	Clearly define all outcomes, exposures, predictors, potential confounders, and effect modifiers. Give diagnostic criteria, if applicable	none
Data sources / measurement	#8	For each variable of interest give sources of data and details of methods of assessment (measurement). Describe comparability of assessment methods if there is more than one group. Give information separately for for exposed and unexposed groups if applicable.	P8-P11
Bias	#9	Describe any efforts to address potential sources of bias	P10-P11
Study size	#10	Explain how the study size was arrived at	P11
Quantitative variables	#11	Explain how quantitative variables were handled in the analyses. If applicable, describe which groupings were chosen, and why	none
Statistical methods	#12a	Describe all statistical methods, including those used to control for confounding	P11
Statistical methods	#12b	Describe any methods used to examine subgroups and interactions	P11
Statistical methods	#12c	Explain how missing data were addressed	none
Statistical methods	#12d	If applicable, describe analytical methods taking account of sampling strategy	none
Statistical methods	#12e	Describe any sensitivity analyses	none

Results

Participants	#13a	Report numbers of individuals at each stage of study—eg numbers potentially eligible, examined for eligibility, confirmed eligible, included in the study, completing follow-up, and analysed. Give information separately for for exposed and unexposed groups if applicable.	P12
Participants	#13b	Give reasons for non-participation at each stage	none
Participants	#13c	Consider use of a flow diagram	none
Descriptive data	#14a	Give characteristics of study participants (eg demographic, clinical, social) and information on exposures and potential confounders. Give information separately for exposed and unexposed groups if applicable.	P12
Descriptive data	#14b	Indicate number of participants with missing data for each variable of interest	P12-P13
Outcome data	#15	Report numbers of outcome events or summary measures. Give information separately for exposed and unexposed groups if applicable.	P12-P14
Main results	#16a	Give unadjusted estimates and, if applicable, confounder-adjusted estimates and their precision (eg, 95% confidence interval). Make clear which confounders were adjusted for and why they were included	P11-P14
Main results	#16b	Report category boundaries when continuous variables were categorized	P11-P14
Main results	#16c	If relevant, consider translating estimates of relative risk into absolute risk for a meaningful time period	none
Other analyses	#17	Report other analyses done—e.g., analyses of subgroups and interactions, and sensitivity analyses	none

Discussion

Key results	#18	Summarise key results with reference to study objectives	P15-P16

Limitations	#19	Discuss limitations of the study, taking into account sources of potential bias or imprecision. Discuss both direction and magnitude of any potential bias.	P16
Interpretation	#20	Give a cautious overall interpretation considering objectives, limitations, multiplicity of analyses, results from similar studies, and other relevant evidence.	P15-P17
Generalisability	#21	Discuss the generalisability (external validity) of the study results	P15-P17
**Other Information**			
Funding	#22	Give the source of funding and the role of the funders for the present study and, if applicable, for the original study on which the present article is based	In title page

None The STROBE checklist is distributed under the terms of the Creative Commons Attribution License CC-BY. This checklist can be completed online using https://www.goodreports.org/, a tool made by the EQUATOR Network in collaboration with Penelope.ai
